# A glycan biomarker predicts cognitive decline in amyloid- and tau-negative patients

**DOI:** 10.1093/braincomms/fcae371

**Published:** 2024-10-18

**Authors:** Robin Ziyue Zhou, Frida Duell, Michael Axenhus, Linus Jönsson, Bengt Winblad, Lars O Tjernberg, Sophia Schedin-Weiss

**Affiliations:** Division of Neurogeriatrics, Department of Neurobiology, Care Sciences and Society, Center for Alzheimer Research, Karolinska Institutet, Solna 171 64, Sweden; Division of Neurogeriatrics, Department of Neurobiology, Care Sciences and Society, Center for Alzheimer Research, Karolinska Institutet, Solna 171 64, Sweden; Division of Neurogeriatrics, Department of Neurobiology, Care Sciences and Society, Center for Alzheimer Research, Karolinska Institutet, Solna 171 64, Sweden; Division of Neurogeriatrics, Department of Neurobiology, Care Sciences and Society, Center for Alzheimer Research, Karolinska Institutet, Solna 171 64, Sweden; Division of Neurogeriatrics, Department of Neurobiology, Care Sciences and Society, Center for Alzheimer Research, Karolinska Institutet, Solna 171 64, Sweden; Theme Inflammation and Aging, Karolinska University Hospital, Huddinge 141 57, Sweden; Division of Neurogeriatrics, Department of Neurobiology, Care Sciences and Society, Center for Alzheimer Research, Karolinska Institutet, Solna 171 64, Sweden; Division of Neurogeriatrics, Department of Neurobiology, Care Sciences and Society, Center for Alzheimer Research, Karolinska Institutet, Solna 171 64, Sweden

**Keywords:** Alzheimer’s disease, biomarkers, CSF, disease progression, N-glycosylation

## Abstract

Early detection of Alzheimer’s disease is vital for timely treatment. Existing biomarkers for Alzheimer’s disease reflect amyloid- and tau-related pathology, but it is unknown whether the disease can be detected before cerebral amyloidosis is observed. N-glycosylation has been suggested as an upstream regulator of both amyloid and tau pathology, and levels of the N-glycan structure bisecting N-acetylglucosamine (GlcNAc) correlate with tau in blood and CSF already at pre-clinical stages of the disease. Therefore, we aimed to evaluate whether bisecting GlcNAc could predict future cognitive decline in patients from a memory clinic cohort, stratified by amyloid/tau status. We included 251 patients (mean age: 65.6 ± 10.6 years, 60.6% female) in the GEDOC cohort, from the Memory Clinic at Karolinska University Hospital, Stockholm, Sweden. Patients were classified as amyloid/tau positive or negative based on CSF biomarkers. Cognitive decline, measured by longitudinal Mini-Mental State Examination scores, was followed for an average of 10.7 ± 4.1 years and modelled using non-linear mixed effects models. Additionally, bisecting GlcNAc levels were measured in hippocampus and cortex with lectin-based immunohistochemistry in 10 Alzheimer’s disease and control brains. We found that CSF bisecting GlcNAc levels were elevated in tau-positive individuals compared with tau-negative individuals, but not in amyloid-positive individuals compared with amyloid-negative individuals. In the whole sample, high levels of CSF bisecting GlcNAc predicted earlier cognitive decline. Strikingly, amyloid/tau stratification showed that high CSF bisecting GlcNAc levels predicted earlier cognitive decline in amyloid-negative patients (*β* = 2.53 ± 0.85 years, *P* = 0.003) and tau-negative patients (*β* = 2.43 ± 1.01 years, *P* = 0.017), but not in amyloid- or tau-positive patients. Finally, histochemical analysis of bisecting GlcNAc showed increased levels in neurons in hippocampus and cortex of Alzheimer’s disease compared with control brain (fold change = 1.44–1.49, *P* < 0.001). In conclusion, high CSF levels of bisecting GlcNAc reflected neuronal pathology and predicted cognitive decline in amyloid- and tau-negative individuals, suggesting that abnormal glycosylation precedes cerebral amyloidosis and tau hyper-phosphorylation in Alzheimer’s disease. Bisecting GlcNAc is a promising novel early biomarker for Alzheimer’s disease.

## Teaser

Zhou *et al*. report that elevated levels of bisecting N-acetylglucosamine–containing N-glycans in CSF could predict cognitive decline in amyloid- and tau-negative memory clinic patients. The authors suggest that bisecting N-acetylglucosamine is an early biomarker in Alzheimer’s disease, which may be altered before amyloid and tau abnormality.

## Graphical Abstract

**Graphical Abstract:**
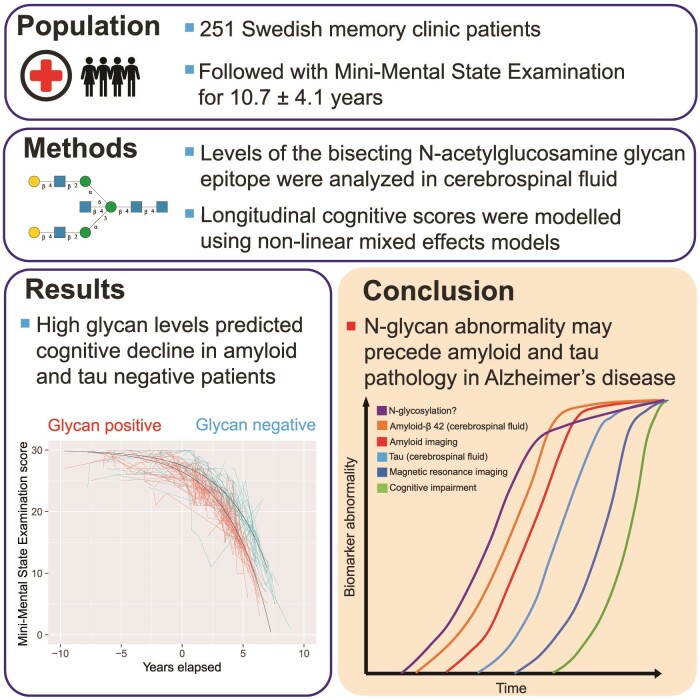

## Introduction

As novel therapies for Alzheimer’s disease have shown effect in slowing the disease course, reliable diagnosis of early Alzheimer’s disease is crucial to select correct patients for treatment.^1,2^ Therefore, biomarkers reflecting Alzheimer’s disease pathophysiology have started to play an ever-increasing role both in research and in the clinical diagnosis of patients. Previously, the temporal course of biomarker abnormality in Alzheimer’s disease has been described. First, an increased accumulation and aggregation of amyloid β-peptide (Aβ) causes amyloid formation in the brain. This precedes the spread of tau aggregates, followed by neurodegeneration and cognitive decline.^3-7^ These features of the disease are reflected by the ATN (amyloid, tau, neurodegeneration) classification system introduced by the National Institute on Aging and Alzheimer’s Association, aiming to classify Alzheimer’s disease on a biological basis.^8^ In this system, biomarkers of different modalities represent three different pathological processes in Alzheimer’s disease—Aβ plaques (A), phosphorylation of tau (T) and neurodegeneration (N).

However, since the factors that precede or initiate aggregation of Aβ and phosphorylation of tau in sporadic Alzheimer’s disease cases are still incompletely known, it is possible that Alzheimer’s disease can be detected before the onset of cerebral amyloidosis and ATN positivity. Since protein N-glycosylation has been implicated in several early pathogenic pathways in Alzheimer’s disease, N-glycosylation patterns should be studied as an early Alzheimer’s disease biomarker.^9-11^ Indeed, previous glycomics studies conducted using mass spectrometry techniques have shown altered N-glycosylation patterns in CSF and brain from patients with Alzheimer’s disease compared with non-demented subjects.^12,13^

**Figure 1:**
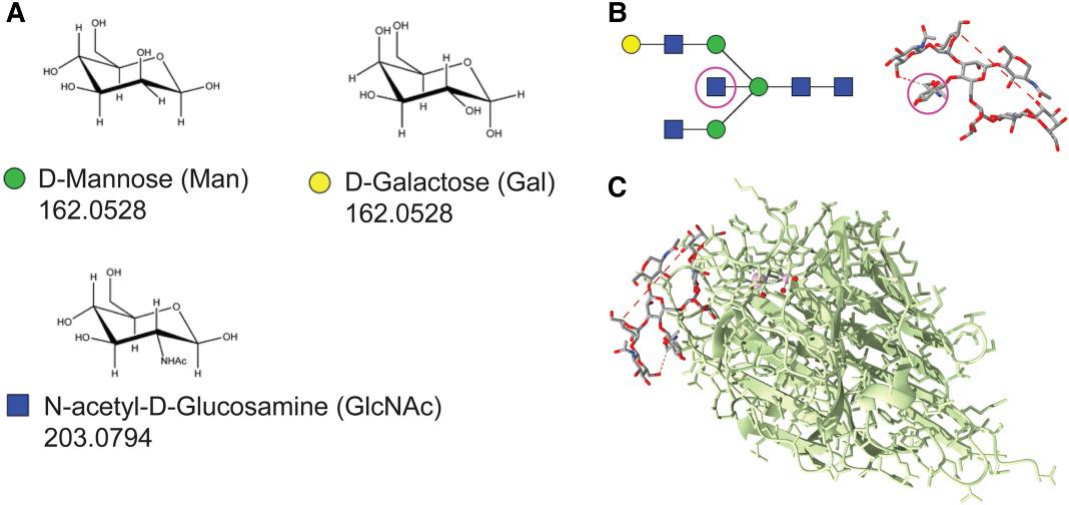
**Structure of bisecting N-acetylglucosamine (GlcNAc) epitope and a model of its complex with PHA-E.** (**A**) Commonly occurring monosaccharides in N-linked glycans. Molecular weights are shown in Dalton. (**B**) Example of a bisecting GlcNAc-containing N-glycan shown as a cartoon drawing and as a molecular model, with the structure itself circled in magenta. (**C**) Model of PHA-E (green) binding to bisecting GlcNAc based on crystallization and X-ray diffraction experiments. One sub-unit of the tetrameric PHA-E protein is shown.

One specific N-glycan structure of interest in Alzheimer’s disease is the bisecting N-acetylglucosamine (GlcNAc) motif. This structure is characterized by a β1–4 linkage between a terminal GlcNAc fragment and a core mannose fragment, which also binds to two other branches of either single saccharide units or longer glycan chains, typically referred to as antennae.^14^ A schematic drawing of bisecting GlcNAc is shown in Fig. 1A and B. Interestingly, bisecting GlcNAc is highly prevalent in brain and has been detected as a post-translational modification on β-site amyloid precursor protein cleaving enzyme 1 (BACE1), influencing its intra-cellular localization and preventing its degradation.^10,15^ Since BACE1 is vital for Aβ production from the amyloid precursor protein, the presence of bisecting GlcNAc may play an early role in Alzheimer’s disease pathogenesis by preserving BACE1 activity and increasing Aβ production within the CNS.^16^

To measure bisecting GlcNAc levels in blood or CSF, we developed a novel high-throughput assay.^17^ The assay is based on the specific binding of the *Phaseolus vulgaris* erythroagglutinin (PHA-E) lectin to the bisecting GlcNAc structure, shown in Fig. 1C.^18^ Using this method, we previously showed that bisecting GlcNAc is elevated in CSF in patients with Alzheimer’s disease.^17^ Importantly, glycan levels correlated with CSF levels of total tau (tTau) and phosphorylated tau-181 (pTau181) already in individuals with subjective cognitive impairment (SCI). The correlation between glycan and tTau values was replicated in blood in a cohort of cognitively normal elderly individuals. Additionally, the bisecting GlcNAc to tTau ratio in blood could predict later progression to Alzheimer’s disease.^19^

Since bisecting GlcNAc levels in blood and CSF correlated to pTau181 and tTau levels already at a pre-clinical stage of Alzheimer’s disease, we hypothesized that bisecting GlcNAc levels may be altered early in the disease course. To evaluate bisecting GlcNAc as an early Alzheimer’s disease biomarker, we analysed glycan levels in CSF of patients in different clinical stages of dementia disease and with different amyloid/tau status. To further link bisecting GlcNAc levels with Alzheimer’s disease–related neurodegeneration, we compared the cognitive trajectory of patients with high or low levels of CSF bisecting GlcNAc.

## Materials and methods

### Study cohort

For this study, 251 patients [99 (39.4%) male, 152 (60.6%) female, mean age of 65.6 ± 10.6 years] were included from the GEDOC research database and biobank. The patients were selected from the SCI, mild cognitive impairment (MCI) and Alzheimer’s disease diagnosis groups. GEDOC contains data and biological material from patients who have visited the memory clinics at Karolinska University Hospital, Stockholm, Sweden. In this cohort, dementia diagnoses followed the guidelines outlined in the Diagnostic and Statistical Manual of Mental Disorders, 4th edition. MCI diagnosis was made based on established criteria requiring the presence of both self-reported and observable cognitive decline in one or more cognitive domains, but no signs of dementia or impairments in daily life activities.^20^

The clinical and demographical data were acquired from the GEDOC database, and a complementary chart review of all patients’ medical history was performed in the electronic medical record software TakeCare by two medical doctors (F.D. and M.A.). The chart review included initial and follow-up visits to identify and collect information about demographics, diagnosis and clinical characteristics at the time of sampling. Subsequently, patients were re-characterized into five disease groups: SCI, MCI, MCI with conversion to Alzheimer’s disease, Alzheimer’s disease and other dementia or MCI with conversion to other dementias. CSF samples were collected from November 2009 to June 2015. All samples were handled according to the consensus guidelines.^21^

### Biomarker measurement

Baseline levels of Aβ42, tTau and pTau181 in CSF were measured at the Clinical Chemistry laboratory of Karolinska University Hospital, Stockholm, Sweden, using a solid-phase enzyme immunoassay on an INNOTEST instrument (Fujirebio Diagnostics, Malvern, PA, USA). Amyloid and tau positivity of individuals were determined by CSF Aβ42 and pTau181 levels, respectively. Clinical cut-off values were used (Aβ42 > 599 ng/L, pTau181 < 56.5 ng/L).

Levels of the bisecting GlcNAc structure were previously measured using a lectin-based assay developed in-house.^17^ For each experiment, glycan levels were normalized against the glycan level of a pooled sample containing CSF from all individuals. The CSF bisecting GlcNAc level of an individual patient is expressed as a ratio to the pooled material. Individuals with higher bisecting GlcNAc levels than the pooled sample (ratio > 1) were considered bisecting GlcNAc positive.

### Brain samples

Brain samples of patients with Alzheimer’s disease and healthy controls, pre-mounted on slides and paraffinized, were obtained from the Netherlands Brain Bank (*n* = 10 for each category). Alzheimer’s disease brain samples met the criteria for definitive Alzheimer’s disease as established by the Consortium to Establish a Registry for Alzheimer’s disease.^22^ The healthy controls had no medical history of psychiatric or neurological disorders. The samples are described in detail in Supplementary Table 1. All samples were cut from whole tissue sections.

### Immunohistochemistry

Brain sections were deparaffinized and hydrated, first in xylene and then in ethanol and water using 99.5, 95 and 70% ethanol w/v with a final bath of distilled water (dH_2_O). Autoclavation in a DIVA decloaker bath at 110°C for 20 min was used for antigen retrieval before a wash with dH_2_O. The protocol for the immunohistochemistry (IHC) kit Envision+ MACH1 universal labelling system (BioCare Medical, CA, USA) was used as follows. Peroxidase blocking of slides was performed in room temperature (RT) for 10 min before a wash with phosphate buffered saline containing 0.05% Tween20, three times for 5 min each while rocking. Blocking was performed with normal goat serum at RT for 30 min. Overnight incubation was performed at 4°C with biotinylated PHA-E lectin, B-1125-2, 1:200 (Vector Laboratories, Newark, CA, USA) in phosphate buffered saline + 4% normal goat serum. As a control, samples were incubated without lectin. After incubation, slides were washed three times for 5 min in phosphate buffered saline containing 0.05% Tween20 followed by secondary labelling with horseradish peroxidase–conjugated streptavidin for 2 h at RT. After secondary incubation, samples were washed with phosphate buffered saline containing 0.05% Tween20, three times for 5 min. Last, slides were incubated with 3,3′-Diaminobenzidine (DAB)-Chromogen solution for 5 min at RT. Slides were rinsed with dH_2_O and dehydrated in a rising concentration of ethanol and with a xylene bath before mounting. A xylene-based mounting medium, Vectamount H5000 (Thermofisher, Waltham, MA, USA), was used. Slides were covered with cover slip panels with a refractive index of 1 and observed using light microscopy within 48 h of mounting. Samples were stored dry and dark when not in use.

### Image acquisition and analysis

IHC pictures of DAB-Chromogen–stained brain sections were captured using a Nikon Camera DS-Qi2 (Nikon, Tokyo, Japan) with capture software Nikon NIS elements. Colour correction channels were adjusted for whitening, and conditions were kept the same for each image capture. The cortex, as well as areas in the hippocampus such as the dentate gyrus (DG) and cornu ammonis 3 (CA3), were imaged and analysed. Images were captured using 10×, 20× and 40× objectives.

Quantification of IHC images was performed using the ImageJ imaging software (NIH, UK). Sub-area signal was defined as all positive signals within the anatomically identified area. Neuronal signal was defined as positivity within neurons identified via morphology. The average signal of 40 neurons per sub-area was used to quantify neuronal signal. The signal intensity was calculated in arbitrary units (AUs) within an interval of 0–250. Unpaired two-tailed Student’s *t*-tests were used to determine if the difference in signal intensities between conditions were significant at an alpha of 0.05.

### Ethical clearance

All participants in the GEDOC cohort provided informed consent for biological samples to be used for future research. The study was conducted according to principles set by the Declaration of Helsinki. Ethical permit for glycan analysis in CSF and human post-mortem brain tissue was obtained from the Ethical Review Board in Stockholm (permit no. 2013/1301-31/2).

### Statistical analysis

Patient data are presented for each disease category (SCI, MCI, other dementia or MCI converting to other dementias, MCI converting to Alzheimer’s disease and Alzheimer’s disease) with mean, median, minimum and maximum values. Numerical variables were compared between groups using Kruskal–Wallis test, since normality was not fulfilled for any variable according to Shapiro–Wilk test using an alpha of 0.05. Categorical variables were compared between groups using Fisher’s exact test. For comparison of bisecting GlcNAc values in different amyloid/tau categories, Kruskal–Wallis test with Dunn’s multiple comparisons test was used. Two-tailed Mann–Whitney U-test was used to compare bisecting GlcNAc values in A+/A− or T+/T− sub-groups.

For modelling of cognitive decline in patients, longitudinal Mini-Mental State Examination (MMSE) scores were used. Patients with no data on MMSE scores were excluded from analysis. To better represent the disease progression in which patients remain relatively stable until a period of accelerating deterioration, we chose to model MMSE decline using a non-linear mixed effects model assuming a left asymptote and exponential MMSE decline.^23^ To account for the fact that patients entered the study at different disease stages, we included a random effect in the form of a random time shift for each individual. The formula for the non-linear model was as follows:


(1) $$30-\mathrm{MMSE}=A\times \exp\;[\alpha (t+\;s_{i})]$$

Where 30−MMSE transforms the measure for cognition into a scale starting at 0 with higher values indicating worse impairment, *A* is scaling parameter of the exponential function, *α* is a scaling parameter of time, *t* is time from initial diagnosis and *s_i_* is an individual time shift. *A* and α were considered fixed effects, while *s_i_* was considered an individual-level random effect.

We compared this model with a linear mixed effects model with a random intercept on individual level, fitted using restricted maximum likelihood estimation. The formula for the linear mixed effects model was as follows:


(2) $$30-\mathrm{MMSE}=\beta_{0}+\beta_{1}t+s_{i}\;$$

Where *β_0_* is the global intercept for the linear model, *β_1_* is the fixed effect parameter for time and *s_i_* is an individual-level random effect. Compared with this linear model, the non-linear model provided better fit of the data according to log-likelihood, Akaike information criterion and Bayesian information criterion parameters. Thus, the non-linear model was used in further analyses. A comparison between the linear mixed effects model and the non-linear mixed effects model is shown in Supplementary Table 2.

To estimate the effect of glycan positivity on cognitive trajectory, we included glycan status as a fixed effect in the non-linear model. The final model is described as follows:


(3) $$30-\mathrm{MMSE}=A\times \exp\;[\alpha (t+\;s_{i}+\;\beta \times G)]$$

Here, *G* is a dummy parameter, which has the value of 1 for glycan-positive individuals and 0 for glycan-negative individuals, and *β* is a fixed effect parameter that models the difference in disease progression for glycan-positive and glycan-negative individuals. Model parameters were fitted using maximum likelihood estimation. Estimation of fixed effects is presented as ±SE. *P*-values for estimates were calculated using Wald’s test. An alpha of 0.05 was used. MMSE progression modelling was performed on the whole sample, as well as in sub-groups including only amyloid/tau positive or negative individuals.

All analyses and preparation of graphs were completed with R version 4.2.0 (r-project.org; R Foundation for Statistical Computing, Vienna, Austria) or GraphPad Prism, version 9.0.0 for Windows (www.graphpad.com; GraphPad Software, San Diego, CA, USA). For non-linear mixed models, the ‘nlme’ package in R was used. For linear mixed models, the ‘lme4’ package was used. For data processing and graphing, the ‘readxl’, ‘tidyr’, ‘stargazer’, ‘dplyr’ and ‘ggplot2’ packages were used.

### Glycan visualization

Drawings of monosaccharides and glycan in Fig. 1 were created in GlycanBuilder and GlycoWorkBench^24^ or modified from a previous publication with the publisher’s permission.^12^ PHA-E crystallization structure was taken from RCSB PDB (PDB code: 5AVA).^18,25^ The molecular model was modified in UCSF ChimeraX, version 1.4 for Mac, developed by the Resource for Biocomputing, Visualization, and Informatics at the University of California, San Francisco.^26^

## Results

Demographic data and biomarker levels for the study population are shown in Table 1. Disease groups were generally congruent with amyloid/tau status, with 62.3% of patients classified as Alzheimer’s disease being amyloid positive (A+) and 81.1% being tau positive (T+). In the SCI group, no individuals were A+, while 21.4% were T+. CSF bisecting GlcNAc levels were measured using a lectin-based assay.^17^ Levels of bisecting GlcNAc increased along the Alzheimer’s disease continuum [SCI: 1.02 ± 0.43 (mean ± SD); MCI: 1.08 ± 0.43; MCI converting to Alzheimer’s disease: 1.15 ± 0.46; Alzheimer’s disease: 1.22 ± 0.42; other dementia or MCI converting to other dementias: 1.37 ± 0.48; Kruskal–Wallis: *P* = 0.005]. Levels of CSF Aβ42, pTau181, tTau and bisecting GlcNAc in different disease categories are visualized in Supplementary Fig. 1.

**Table 1:** Demographics of the study population

		MCI (*n* = 75)				
	Alzheimer’s disease (*n* = 106)	Converting to Alzheimer’s disease (*n* = 37)	No conversion during follow-up (*n* = 27)	Converting to other dementia (*n* = 11)^a^	SCI (*n* = 70)	*P*-value^b^
Age (years)						
Mean (SD)	71.0 (9.24)	68.8 (7.98)	60.7 (6.99)	70.0 (10.6)	56.8 (8.07)	
Median (min, max)	72 (50, 89)	69 (54, 85)	61 (48, 77)	70 (52, 85)	57.5 (37, 79)	<0.001
Sex						
Male	34 (32.1%)	13 (35.1%)	14 (51.9%)	8 (72.7%)	30 (42.9%)	
Female	72 (67.9%)	24 (64.9%)	13 (48.1%)	3 (27.3%)	40 (57.1%)	0.045
Follow-up time (months)						
Mean (SD)	105 (50.6)	149 (47.1)	147 (36.7)	115 (37.8)	149 (38.5)	
Median (min, max)	91 (20, 191)	157 (24, 191)	157 (50, 191)	116 (34, 191)	131 (70, 191)	<0.001
Conversion time (months)						
Mean (SD)		28.2 (22.2)		34.2 (31.1)		
Median (min, max)		21 (5, 97)		17 (7, 84)		0.917
Missing		0 (0%)		1 (9.1%)		
*APOE4* ^c^ carriership						
At least one *APOE4* allele	40 (37.7%)	20 (54.1%)	6 (22.2%)	1 (9.1%)	25 (35.7%)	
No *APOE4* allele	18 (17.0%)	6 (16.2%)	10 (37.0%)	4 (36.4%)	24 (34.3%)	0.009
Missing	48 (45.3%)	11 (29.7%)	11 (40.7%)	6 (54.5%)	21 (30.0%)	
Baseline MMSE						
Mean (SD)	22.6 (4.32)	26.6 (1.97)	26.8 (2.75)	28.2 (1.17)	28.9 (1.45)	
Median (min, max)	23 (10, 30)	27 (21, 30)	27 (20, 30)	28 (26, 30)	29 (22, 30)	<0.001
Missing	2 (1.9%)	2 (5.4%)	3 (11.1%)	0 (0%)	5 (7.1%)	
A+						
Yes	66 (62.3%)	5 (18.5%)	19 (51.4%)	2 (18.2%)	0 (0%)	
No	34 (32.1%)	20 (74.1%)	17 (45.9%)	9 (81.8%)	61 (87.1%)	<0.001
Missing	6 (5.7%)	2 (7.4%)	1 (2.7%)	0 (0%)	9 (12.9%)	
T+						
Yes	86 (81.1%)	7 (25.9%)	33 (89.2%)	4 (36.4%)	15 (21.4%)	
No	14 (13.2%)	18 (66.7%)	3 (8.1%)	7 (63.6%)	46 (65.7%)	<0.001
Missing	6 (5.7%)	2 (7.4%)	1 (2.7%)	0 (0%)	9 (12.9%)	
CSF tTau (ng/L)						
Mean (SD)	688 (306)	597 (166)	275 (84.2)	369 (126)	258 (140)	
Median (min, max)	677 (154, 1910)	594 (240, 949)	264 (152, 449)	325 (186, 628)	218 (97.0, 681)	<0.001
Missing	6 (5.7%)	1 (2.7%)	2 (7.4%)	0 (0%)	9 (12.9%)	
CSF pTau (ng/L)						
Mean (SD)	89.1 (33.0)	82.8 (21.5)	49.3 (10.8)	55.0 (12.8)	47.9 (20.9)	
Median (min, max)	86 (27, 221)	80 (29, 130)	48 (29, 69)	55 (33, 72)	43 (10, 103)	<0.001
Missing	6 (5.7%)	1 (2.7%)	2 (7.4%)	0 (0%)	9 (12.9%)	
CSF Aβ (1–42) (ng/L)						
Mean (SD)	559 (129)	631 (244)	983 (346)	986 (345)	1150 (274)	
Median (min, max)	565 (271, 958)	580 (383, 1550)	1030 (406, 1530)	1050 (357, 1490)	1150 (621, 2030)	<0.001
Missing	6 (5.7%)	1 (2.7%)	2 (7.4%)	0 (0%)	9 (12.9%)	
CSF bisecting GlcNAc (relative levels)						
Mean (SD)	1.22 (0.420)	1.15 (0.464)	1.08 (0.432)	1.37 (0.480)	1.02 (0.432)	
Median (min, max)	1.12 (0.502, 2.65)	1.03 (0.487, 2.52)	1.07 (0.443, 2.68)	1.44 (0.470, 2.17)	0.856 (0.412, 2.21)	0.005
Missing	10 (9.4%)	1 (2.7%)	2 (7.4%)	0 (0%)	2 (2.9%)	

^a^One patient in this group was classified as ‘other dementia’ already at baseline.

^b^Calculated using Fisher’s exact test for categorical variables and Kruskal–Wallis test for numerical variables.

^c^Apolipoprotein E ε4 allele.

**Figure 2:**
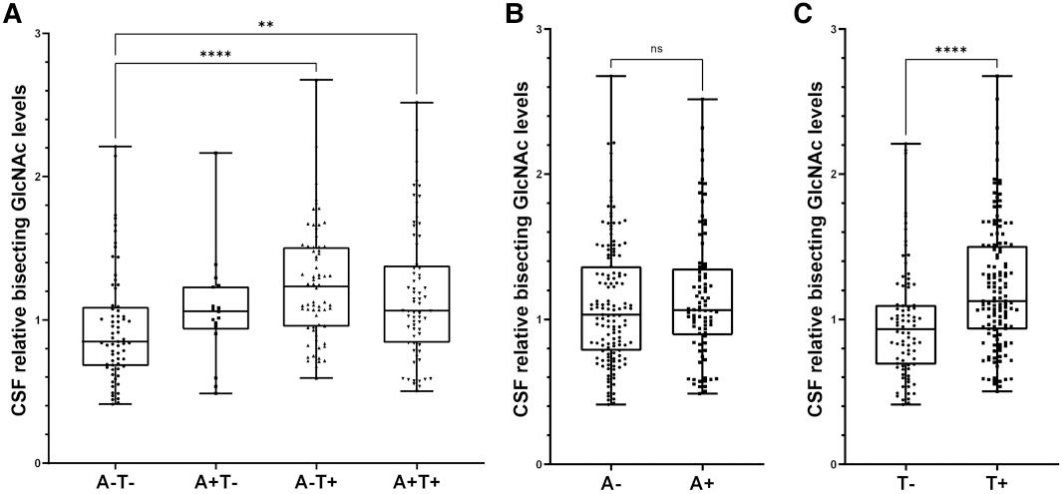
**CSF bisecting N-acetylglucosamine (GlcNAc) levels in different amyloid/tau sub-groups. (A)** CSF bisecting GlcNAc levels were plotted for different amyloid/tau sub-groups. Groups were compared using Kruskal–Wallis test adjusted for multiple comparisons. The number of individuals in each group was *n* = 67 in the A−T− group, *n* = 17 in the A+T− group, *n* = 68 in the A−T+ group and *n* = 67 in the A+T+ group. **(B** and **C)** CSF bisecting GlcNAc levels in A+/A− or T+/T− patients. Groups were compared using two-tailed Mann–Whitney U-test. The number of individuals in each group were *n* = 135 in the A− group, *n* = 84 in the A+ group, *n* = 84 in the T− group and *n* = 135 in the T+ group. In all graphs, all data points are shown. Boxes indicate median and 25th/75th percentile; whiskers indicate maximum and minimum values. Significances shown: **P < 0.01, ****P < 0.0001. A, amyloid; T, tau.

When comparing CSF bisecting GlcNAc levels in different amyloid/tau sub-groups, bisecting GlcNAc was significantly elevated in the A−T+ and A+T+ groups compared with A−T− patients [A−T−: 0.95 ± 0.40 (mean ± SD); A+T−: 1.07 ± 0.38; A−T+: 1.25 ± 0.40; A+T+: 1.18 ± 0.46; A−T− versus A−T+: *P* < 0.001; A−T− versus A+T+: *P* = 0.006]. Interestingly, CSF bisecting GlcNAc was elevated in T+ individuals compared with T− individuals [T−: 0.97 ± 0.39 (mean ± SD); T+: 1.21 ± 0.43; *P* < 0.001], but not in A+ individuals compared with A− individuals (A−: 1.10 ± 0.42; A+: 1.16 ± 0.45; *P* = 0.39). This is in agreement with our previous results, showing that CSF bisecting GlcNAc correlates with CSF pTau181 but not with CSF Aβ42.^17^ Graphical visualization of CSF bisecting GlcNAc levels in different A/T sub-groups is shown in Fig. 2.

For analysis of global cognitive decline, we modelled MMSE score progression using a non-linear mixed effects model. Clinical data including MMSE were extracted from individual patient files, from the time of initial diagnosis to an average of 10.7 ± 4.1 years (mean ± SD) afterward. Individual MMSE trajectories from the date of initial diagnosis, stratified by disease group, are shown in Supplementary Fig. 2a. By allowing for a random patient-level shift, all MMSE trajectories can be fitted to one model based on a non-linear relationship between MMSE and time, regardless of baseline MMSE. As a result, a common pattern for cognitive decline spanning over 15 years can be aligned for the entire cohort, as seen in Supplementary Fig. 2b.

**Figure 3:**
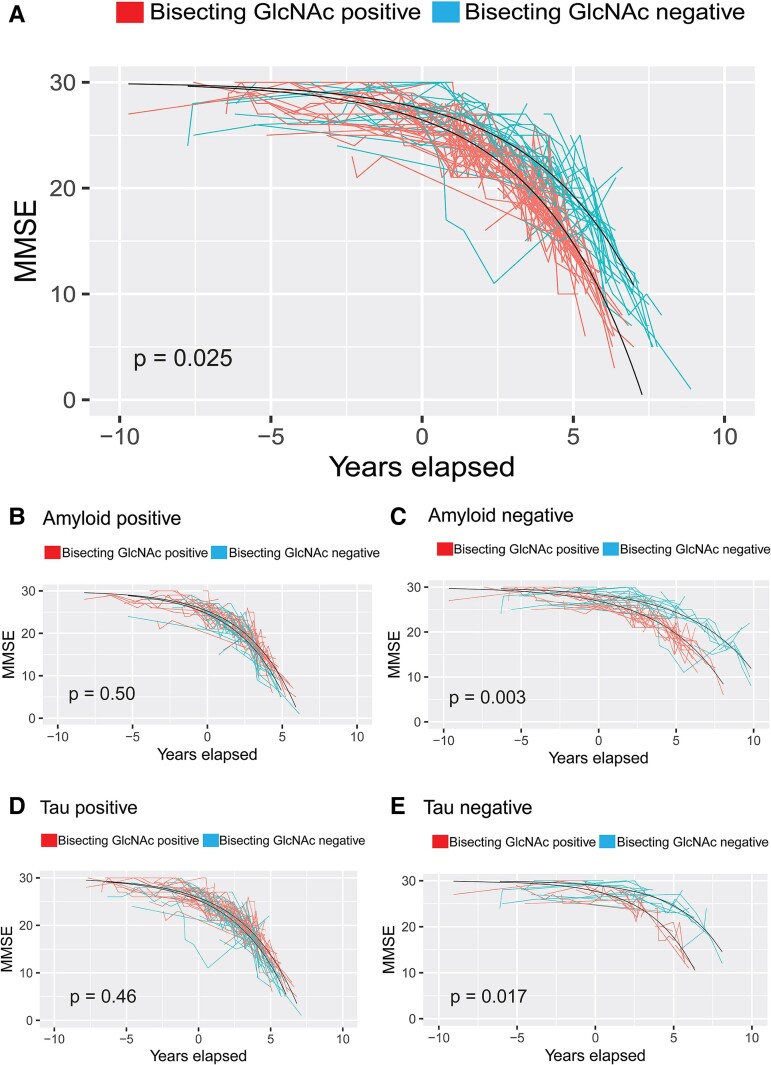
**The effect of bisecting N-acetylglucosamine (GlcNAc) positivity on cognitive decline. (A)** MMSE trajectories of patients stratified by CSF baseline levels of bisecting N-acetylglucosamine glycan in the whole GEDOC sample. MMSE data based on *n* = 814 observations in *n* = 228 individuals. Degrees of freedom (*df*) of non-linear model = 584. **(B** and **C)** MMSE trajectories of amyloid-positive or amyloid-negative patients stratified by glycan levels. Data based on *n* = 380 observations in *n* = 80 individuals in the amyloid-positive group (*df* = 298) and *n* = 395 observations in *n* = 132 individuals in the amyloid-negative group (*df* = 261). **(D** and **E)** MMSE trajectories of tau-positive or tau-negative patients stratified by glycan levels. Data based on *n* = 583 observations in *n* = 132 individuals in the tau-positive group (*df* = 449) and *n* = 192 observations in *n* = 80 patients in the tau-negative group (*df* = 110). In all graphs, *P*-values were calculated by applying Wald’s test on the estimate of the fixed effect of glycan positivity in a non-linear mixed effects model.

To examine whether CSF bisecting GlcNAc levels could predict cognitive decline, glycan positivity (defined as normalized bisecting GlcNAc level >1.0) was included as a fixed effect in the previously described model. Glycan-positive individuals experienced MMSE decline earlier than glycan-negative individuals [estimated effect (*β*) = 1.20 ± 0.53 years, *P* = 0.025]. As shown in Supplementary Table 3, age and sex were not significant predicting variables in this model and were thus omitted in the analysis. A graphical visualization of MMSE trajectories in glycan-positive or glycan-negative individuals can be seen in Fig. 3A.

To better understand the inter-play between bisecting GlcNAc and amyloid/tau positivity, we then performed the same analysis in sub-groups based on amyloid/tau biomarker status, shown in Fig. 3B–E. In amyloid-positive individuals, glycan-positive or glycan-negative groups did not differ significantly in MMSE trajectory (*β* = −0.45 ± 0.66 years, *P* = 0.50). However, in amyloid-negative individuals, glycan-positive individuals declined earlier in MMSE, with a larger estimated effect size compared with glycan positivity in the whole population (*β* = 2.53 ± 0.85 years, *P* = 0.003). Similarly, glycan positivity predicted MMSE decline in tau-negative individuals (*β* = 2.43 ± 1.01 years, *P* = 0.017), but not in tau-positive individuals (*β* = −0.46 ± 0.62 years, *P* = 0.46).

**Figure 4:**
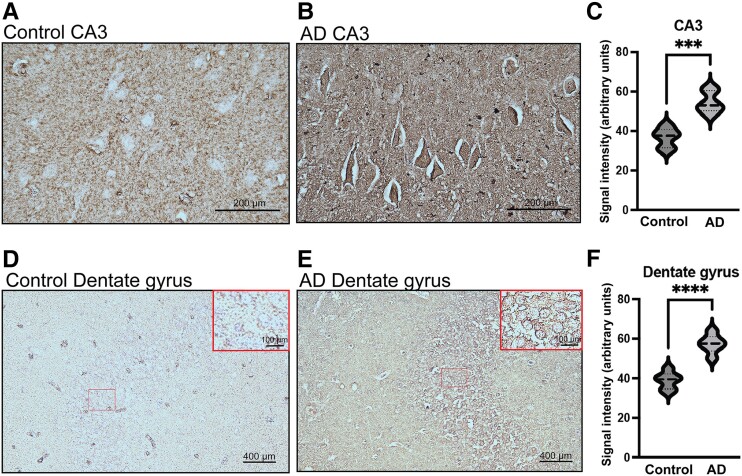
**IHC of PHA-E in Alzheimer’s disease and control hippocampus**. **(A** and **B)** Representative images displaying PHA-E staining in CA3 of Alzheimer’s disease and control brain. **(C)** Quantification of PHA-E signal intensity in CA3 of Alzheimer’s disease compared with control. **(D** and **E)** Representative images displaying PHA-E staining in DG of Alzheimer’s disease and control brain. **(F)** Quantification of PHA-E signal intensity in DG of Alzheimer’s disease compared with control. In **C** and **F**, graphs show signal intensity with lines indicating median, 25th percentile, and 75th percentile. Groups were compared using unpaired two-tailed Student’s *t*-test. Significances shown: ***P < 0.001, ****P < 0.0001.

Finally, we aimed to determine whether the elevated levels of bisecting GlcNAc in CSF of patients with Alzheimer’s disease were reflected in the brain. Brain sections from patients with Alzheimer’s disease and healthy controls were stained using PHA-E lectin (the same lectin as used for CSF quantification), which binds to the bisecting GlcNAc structure. Image analysis showed increased signal intensity in neurons of the hippocampus of Alzheimer’s disease brain compared with control. In CA3, the mean relative signal intensity was 36.7 ± 4.5 AU in control brain and 54.5 ± 5.7 AU in Alzheimer’s disease brain (fold change: 1.49, *P* < 0.001). In the DG, the mean relative signal intensity was 38.8 ± 4.3 AU in control brain and 57.3 ± 4.4 AU in Alzheimer’s disease brain (fold change: 1.48, *P* < 0.001). Furthermore, PHA-E staining intensity was also increased in Alzheimer’s disease cortex compared with control (control: 25.3 ± 2.5 AU, Alzheimer’s disease: 36.5 ± 3.2 AU, fold change: 1.44, *P* < 0.001). Quantification of signal intensity and representative images of hippocampal brain sections are shown in Fig. 4. Representative images of cortical brain sections are shown in Supplementary Fig. 3.

## Discussion

In this study, we showed that high levels of bisecting GlcNAc can predict earlier cognitive decline in amyloid-negative and in tau-negative patients, but not in amyloid-/tau-positive patients. This suggests that elevated levels of bisecting GlcNAc occur early in Alzheimer’s disease pathogenesis, even prior to amyloid/tau abnormality, as it can predict cognitive decline already at an amyloid-/tau-negative stage. Furthermore, since bisecting GlcNAc was elevated in tau-positive individuals compared with tau-negative individuals, we suggest that bisecting GlcNAc abnormality is at least partially temporally associated with tau pathology in Alzheimer’s disease. Last, bisecting GlcNAc was elevated in both CSF and brain in Alzheimer’s disease, linking it to the pathogenesis of the disease.

This paper replicates and expands upon our previous studies describing the apparent relationship among bisecting GlcNAc, tTau and pTau181 in early Alzheimer’s disease.^17,19^ In two separate cohorts, we demonstrated that bisecting GlcNAc correlates with tTau in blood and with pTau181 in CSF in cognitively healthy elderly who are at risk of developing Alzheimer’s disease. Although the mechanism behind this is unknown, previous studies have shown that tau is N-glycosylated in Alzheimer’s disease brain and that bisecting GlcNAc is one of the most common N-glycosylation structures on tau in Alzheimer’s disease.^27,28^ N-glycosylation of tau has been suggested as an upstream modulator of tau phosphorylation.^29,30^ In this context, our study adds strength to the hypothesis that aberrant N-glycosylation of tau may occur earlier than phosphorylation, since the bisecting GlcNAc structure was elevated in tau-positive patients but also could predict earlier cognitive decline in tau-negative patients.

As expected, amyloid- or tau-negative individuals showed slower cognitive decline compared with their amyloid-positive or tau-positive counterparts, but glycan positivity was a significant negative prognostic factor for future cognition in the amyloid-/tau-negative groups. We interpret this as evidence that bisecting GlcNAc positivity precedes amyloid/tau positivity in Alzheimer’s disease. However, using this cohort, we cannot ascertain that amyloid-/tau-negative individuals who are glycan positive later develop Alzheimer’s disease–related pathology, since no longitudinal data on amyloid/tau status are available and no samples from patients with other neurodegenerative diseases were analysed. Although this is a limitation of our study, there are other factors suggesting that bisecting GlcNAc is implicated in Alzheimer’s disease. First, we detected only few cases of other dementias during the follow-up period (*n* = 11). Second, of MCI cases that progressed to dementia, 37 patients progressed to Alzheimer’s disease and 10 patients progressed to other dementias. Finally, IHC studies showed an abundance of bisecting GlcNAc in neurons of Alzheimer’s disease brain. Still, further studies that elucidate the exact disease phenotype of these amyloid-/tau-negative and glycan-positive individuals are needed.

In this study, we used a non-linear mixed effects model for MMSE progression, which reflects the natural disease course. In studies with relatively short follow-up on cognitive scores and a homogenous study population, such as clinical trials for Alzheimer’s disease, the trajectory of cognitive scores seems linear in nature.^1,31,32^ Linear mixed effects models are commonly used to estimate the effect of certain variables on disease progression and may be appropriate in some instances.^33,34^ However, by using a non-linear model with random intercept for each individual, we account for the fact that patients enrol in this study at different stages of the disease and that patients with lower cognitive function also tend to decline faster.^35^ Since our study has a long follow-up time, we expect patients with early Alzheimer’s disease to remain relatively stable before a period of accelerating decline, which suits an exponential MMSE model.

For future Alzheimer’s disease diagnosis, there is growing consensus that Alzheimer’s disease diagnosis should be based on the biological basis of the disease, as evidenced by the ATN framework proposed by the National Institute on Aging and Alzheimer’s Association.^8^ While amyloid/tau positivity is a clear risk factor for developing future dementia,^36^ most persons considered to have Alzheimer’s disease pathology according to the ATN framework do not have dementia.^37^ In order to link bisecting GlcNAc to both the biological definition of the disease and its clinical manifestations, we have included both amyloid/tau category and clinical disease progression as evidenced by worsening MMSE in our analyses. We show that bisecting GlcNAc is a risk factor for dementia development in amyloid-/tau-negative individuals, who are not considered to be at risk for dementia due to Alzheimer’s disease according to the National Institute on Aging and Alzheimer’s Association classification. Thus, we propose that bisecting GlcNAc is a valuable complement to the ATN system and allows risk stratification even in a low-risk amyloid-/tau-negative population. Above all, our study is an important proof of concept of the utility of glycans as a diagnostic tool in early Alzheimer’s disease and the potential for this type of biomarkers to precede amyloid or tau abnormality.

In our study, we measured general levels of the bisecting GlcNAc glycan epitope, without considering the proteins to which they are attached. Our finding that bisecting GlcNAc was generally increased in Alzheimer’s disease is consistent with an upregulation of the sole synthesizing enzyme for this epitope (N-acetylglucosaminyltransferase III), which has been reported to be the case in Alzheimer’s disease.^38^ However, levels of specific glycopeptides may be more specific markers for Alzheimer’s disease pathology. Here, methodological advances in glycomics/glycoproteomics will further advance our understanding of glycosylation as a pathogenic mechanism in Alzheimer’s disease and generate more glycan/glycopeptide candidates as potential biomarkers in Alzheimer’s disease.^39^ Additionally, analysis of glycosylation patterns can aid in dividing the heterogenous disease group of Alzheimer’s disease into different sub-types with distinct biological processes, which each may require tailored therapy options.^40^

## Conclusion

Bisecting GlcNAc is a tau-related marker that is elevated in Alzheimer’s disease both in CSF and brain. The results support previous research suggesting that aberrant N-glycosylation—especially with the bisecting GlcNAc structure—may be an upstream regulator of Aβ production and tau phosphorylation. Bisecting GlcNAc is an early Alzheimer’s disease biomarker, which predicts cognitive decline already at an amyloid-/tau-negative stage.

## Supplementary Material

fcae371_Supplementary_Data

## Data Availability

Anonymized data are available upon reasonable request to the corresponding author, subject to approval by Swedish ethical review authorities. R code used for data analysis and generation of graphs is available in the Supplementary material.

## References

[1] van Dyck CH, Swanson CJ, Aisen P, et al Lecanemab in early Alzheimer’s disease. N Engl J Med. 2023;388(1):9–21. 36449413 10.1056/NEJMoa2212948

[2] Sims JR, Zimmer JA, Evans CD, et al Donanemab in early symptomatic Alzheimer disease. JAMA. 2023;330(6):512. 37459141 10.1001/jama.2023.13239 PMC10352931

[3] Sperling RA, Aisen PS, Beckett LA, et al Toward defining the preclinical stages of Alzheimer’s disease: Recommendations from the National Institute on Aging-Alzheimer’s Association workgroups on diagnostic guidelines for Alzheimer’s disease. Alzheimers Dement. 2011;7(3):280–292. 21514248 10.1016/j.jalz.2011.03.003 PMC3220946

[4] Jack CR, Knopman DS, Jagust WJ, et al Hypothetical model of dynamic biomarkers of the Alzheimer’s pathological cascade. Lancet Neurol. 2010;9(1):119–128. 20083042 10.1016/S1474-4422(09)70299-6 PMC2819840

[5] Jack CR, Vemuri P, Wiste HJ, et al Shapes of the trajectories of 5 major biomarkers of Alzheimer disease. Arch Neurol. 2012;69(7):856–867. 22409939 10.1001/archneurol.2011.3405 PMC3595157

[6] Petersen RC . Alzheimer’s disease: Progress in prediction. Lancet Neurol. 2010;9(1):4–5. 20083022 10.1016/S1474-4422(09)70330-8 PMC3098141

[7] Blennow K, Hampel H, Weiner M, Zetterberg H. Cerebrospinal fluid and plasma biomarkers in Alzheimer disease. Nat Rev Neurol. 2010;6(3):131–144. 20157306 10.1038/nrneurol.2010.4

[8] Jack CR, Bennett DA, Blennow K, et al NIA-AA research framework: Toward a biological definition of Alzheimer’s disease. Alzheimers Dement. 2018;14(4):535–562. 29653606 10.1016/j.jalz.2018.02.018 PMC5958625

[9] Bradberry MM, Peters-Clarke TM, Shishkova E, Chapman ER, Coon JJ. N-glycoproteomics of brain synapses and synaptic vesicles. Cell Rep. 2023;42(4):112368. 37036808 10.1016/j.celrep.2023.112368 PMC10560701

[10] Kizuka Y, Nakano M, Kitazume S, Saito T, Saido TC, Taniguchi N. Bisecting GlcNAc modification stabilizes BACE1 protein under oxidative stress conditions. Biochem J. 2016;473(1):21–30. 26467158 10.1042/BJ20150607

[11] Lin T, van Husen LS, Yu Y, Tjernberg LO, Schedin-Weiss S. Lack of N-glycosylation increases amyloidogenic processing of the amyloid precursor protein. Glycobiology. 2022;32(6):506–517. 35275192 10.1093/glycob/cwac009 PMC9132248

[12] Gaunitz S, Tjernberg LO, Schedin-Weiss S. What can N-glycomics and N-glycoproteomics of cerebrospinal fluid tell us about Alzheimer disease? Biomolecules. 2021;11(6):858. 34207636 10.3390/biom11060858 PMC8226827

[13] Gaunitz S, Tjernberg LO, Schedin-Weiss S. The N-glycan profile in cortex and hippocampus is altered in Alzheimer disease. J Neurochem. 2021;159(2):292–304. 32986846 10.1111/jnc.15202 PMC8596851

[14] Chen Q, Tan Z, Guan F, Ren Y. The essential functions and detection of bisecting GlcNAc in cell biology. Front Chem. 2020;8:511. 32719771 10.3389/fchem.2020.00511 PMC7350706

[15] Kizuka Y, Kitazume S, Fujinawa R, et al An aberrant sugar modification of BACE1 blocks its lysosomal targeting in Alzheimer’s disease. EMBO Mol Med. 2015;7(2):175–189. 25592972 10.15252/emmm.201404438 PMC4328647

[16] Hampel H, Vassar R, De Strooper B, et al The β-secretase BACE1 in Alzheimer’s disease. Biol Psychiatry. 2021;89(8):745–756. 32223911 10.1016/j.biopsych.2020.02.001 PMC7533042

[17] Schedin-Weiss S, Gaunitz S, Sui P, et al Glycan biomarkers for Alzheimer disease correlate with T-tau and P-tau in cerebrospinal fluid in subjective cognitive impairment. FEBS J. 2020;287(15):3221–3234. 31889402 10.1111/febs.15197 PMC7496940

[18] Nagae M, Kanagawa M, Morita-Matsumoto K, et al Atomic visualization of a flipped-back conformation of bisected glycans bound to specific lectins. Sci Rep. 2016;6(1):22973. 26971576 10.1038/srep22973 PMC4789653

[19] Zhou RZ, Vetrano DL, Grande G, et al A glycan epitope correlates with tau in serum and predicts progression to Alzheimer’s disease in combination with APOE4 allele status. Alzheimers Dement. 2023;19(7):3244–3249. 37042462 10.1002/alz.13024

[20] Winblad B, Palmer K, Kivipelto M, et al Mild cognitive impairment—Beyond controversies, towards a consensus: Report of the international working group on mild cognitive impairment. J Intern Med. 2004;256(3):240–246. 15324367 10.1111/j.1365-2796.2004.01380.x

[21] Teunissen CE, Petzold A, Bennett JL, et al A consensus protocol for the standardization of cerebrospinal fluid collection and biobanking. Neurology. 2009;73(22):1914–1922. 19949037 10.1212/WNL.0b013e3181c47cc2 PMC2839806

[22] Moms JC, Heyman A, Mohs RC, et al The Consortium to Establish a Registry for Alzheimer’s Disease (CERAD). Part I. Clinical and neuropsychological assesment of Alzheimer’s disease. Neurology. 1989;39(9):1159–1159. 2771064 10.1212/wnl.39.9.1159

[23] Raket LL . Statistical disease progression modeling in Alzheimer disease. Front Big Data. 2020;3:24. 33693397 10.3389/fdata.2020.00024 PMC7931952

[24] Damerell D, Ceroni A, Maass K, Ranzinger R, Dell A, Haslam SM. The GlycanBuilder and GlycoWorkbench glycoinformatics tools: Updates and new developments. Biol Chem. 2012;393(11):1357–1362. 23109548 10.1515/hsz-2012-0135

[25] Berman HM . The protein data bank. Nucleic Acids Res. 2000;28(1):235–242. 10592235 10.1093/nar/28.1.235 PMC102472

[26] Pettersen EF, Goddard TD, Huang CC, et al UCSF ChimeraX: Structure visualization for researchers, educators, and developers. Protein Sci. 2021;30(1):70–82. 32881101 10.1002/pro.3943 PMC7737788

[27] Sato Y, Naito Y, Grundke-Iqbal I, Iqbal K, Endo T. Analysis of N-glycans of pathological tau: Possible occurrence of aberrant processing of tau in Alzheimer’s disease. FEBS Lett. 2001;496(2–3):152–160. 11356201 10.1016/s0014-5793(01)02421-8

[28] Wang JZ, Grundke-Iqbal I, Iqbal K. Glycosylation of microtubule–associated protein tau: An abnormal posttranslational modification in Alzheimer’s disease. Nat Med. 1996;2(8):871–875. 8705855 10.1038/nm0896-871

[29] Liu F, Zaidi T, Iqbal K, Grundke-Iqbal I, Gong CX. Aberrant glycosylation modulates phosphorylation of tau by protein kinase A and dephosphorylation of tau by protein phosphatase 2A and 5. Neuroscience. 2002;115(3):829–837. 12435421 10.1016/s0306-4522(02)00510-9

[30] Losev Y, Frenkel-Pinter M, Abu-Hussien M, et al Differential effects of putative N-glycosylation sites in human tau on Alzheimer’s disease-related neurodegeneration. Cell Mol Life Sci. 2021;78(5):2231–2245. 32926180 10.1007/s00018-020-03643-3 PMC11072875

[31] Budd Haeberlein S, Aisen PS, Barkhof F, et al Two randomized phase 3 studies of aducanumab in early Alzheimer’s disease. J Prev Alzheimers Dis. 2022;9:197–210. 35542991 10.14283/jpad.2022.30

[32] Mintun MA, Lo AC, Duggan Evans C, et al Donanemab in early Alzheimer’s disease. N Engl J Med. 2021;384(18):1691–1704. 33720637 10.1056/NEJMoa2100708

[33] Mielke MM, Aakre JA, Algeciras-Schimnich A, et al Comparison of CSF phosphorylated tau 181 and 217 for cognitive decline. Alzheimers Dement. 2022;18(4):602–611. 34310832 10.1002/alz.12415 PMC8789950

[34] Katzourou I, Leonenko G, Ivanov D, et al Cognitive decline in Alzheimer’s disease is not associated with APOE. J Alzheimers Dis. 2021;84(1):141–149. 34487047 10.3233/JAD-210685

[35] Gueorguieva I, Chua L, Willis BA, Sims JR, Wessels AM. Disease progression model using the integrated Alzheimer’s disease rating scale. Alzheimers Dement. 2023;19(6):2253–2264. 36450003 10.1002/alz.12876

[36] van Harten AC, Smits LL, Teunissen CE, et al Preclinical AD predicts decline in memory and executive functions in subjective complaints. Neurology. 2013;81(16):1409–1416. 24049134 10.1212/WNL.0b013e3182a8418b

[37] Gustavsson A, Norton N, Fast T, et al Global estimates on the number of persons across the Alzheimer’s disease continuum. Alzheimers Dement. 2023;19(2):658–670. 35652476 10.1002/alz.12694

[38] Akasaka-Manya K, Manya H, Sakurai Y, et al Protective effect of N-glycan bisecting GlcNAc residues on beta-amyloid production in Alzheimer’s disease. Glycobiology. 2010;20(1):99–106. 19776078 10.1093/glycob/cwp152

[39] Riley NM, Hebert AS, Westphall MS, Coon JJ. Capturing site-specific heterogeneity with large-scale N-glycoproteome analysis. Nat Commun. 2019;10(1):1311. 30899004 10.1038/s41467-019-09222-w PMC6428843

[40] Tijms BM, Vromen EM, Mjaavatten O, et al Cerebrospinal fluid proteomics in patients with Alzheimer’s disease reveals five molecular subtypes with distinct genetic risk profiles. Nat Aging. 2024;4(1):33–47. 38195725 10.1038/s43587-023-00550-7 PMC10798889

