# Supplementary material for: A glycan biomarker predicts cognitive decline in amyloid- and tau-negative patients
Source: Brain Commun. 2024 Oct 18;6(6):fcae371. doi: 10.1093/braincomms/fcae371 (PMC11528473; doi:10.1093/braincomms/fcae371)
Supplement: fcae371_Supplementary_Data [file fcae371_supplementary_data.pdf]

Supplementary Material to Zhou RZ et al. A glycan biomarker predicts cognitive decline in amyloid- and tau-negative patients

Table of contents

Supplementary Table 1 Patient information for Alzheimer's disease (AD) and control samples used for immunohistochemistry 2

Supplementary Table 2 Comparison between linear- and non-linear mixed effect models 2

Supplementary Table 3 Non-linear mixed-effects model including bisecting GlcNAc positivity as a fixed effect, adjusted for age or sex 3

Supplementary Figure 1 Levels of CSF biomarkers at baseline in different disease groups 4

Supplementary Figure 2 Cognitive decline as measured by longitudinal Mini-Mental State Examination (MMSE) scores in the GEDOC cohort. 5

Supplementary Figure 3 Immunohistochemistry of Phaseolus vulgaris erythroagglutinin (PHA-E) in Alzheimer's disease (AD) and control cortex. 6

Supplementary Methods: R code used for data analysis and visualization in this study. 7

Supplementary Table 1 Patient information for Alzheimer's disease (AD) and control samples used for immunohistochemistry

		Clinical diagnosis	
		**AD (n = 10)**	**Control (n = 10)**
Age of death (years), mean, SD (range)		82 ± 3.1 (74-88)	83 ± 7.2 (73-91)
Number of females		7	7
Brain weight (g), mean, SD (range)		985 ± 100.8 (843-1135)	1119 ± 122 (1001-1361)
Post-mortem interval (hours), mean, SD (range)		4.2 ± 0.8 (3.2-5.4)	5.3 ± 1.4 (5.1-7.4)
Distribution of Braak scores	0	0	10
	I/II	0	0
	III/IV	0	0
	V/VI	10	0

Supplementary Table 2 Comparison between linear- and non-linear mixed effect models

	Model	
**Variables**	**Linear mixed effects**	**Non-linear mixed effects**
β_1[a]	1.020*** (0.067)	
A[b]		3.123*** (0.238)
α[c]		0.293*** (0.009)
β_0[d]	5.038*** (0.341)	
Observations	875	875
Log Likelihood[e]	-2547.019	-2404.137
Akaike Information Criterion[f]	5102.038	4816.274
Bayesian Information Criterion[g]	5121.135	4835.371

The dependent variable was 30-MMSE in both models. Time was measured in years. All parameter estimates are presented as mean ± standard error.

[a] Fixed effect parameter for time in the linear model.
[b] Scaling factor for the exponential model.
[c] Scaling factor for time in the exponential model.
[d] Global intercept for the linear model.
[e] Higher number indicate better model fit.
[f, g] Lower number indicate better model fit.
* $p < 0.05$, ** $p < 0.01$, *** $p < 0.001$

Supplementary Table 3 Non-linear mixed-effects model including bisecting GlcNAc positivity as a fixed effect, adjusted for age or sex

	Model		
**Variables**	**Base model**	**Adjusted for sex**	**Adjusted for age**
A[a]	2.507***	2.271***	2.257
	(0.321)	(0.377)	(1.181)
α[b]	0.291***	0.291***	0.289***
	(0.010)	(0.010)	(0.013)
β_1[c]	1.202*	1.264*	1.163*
	(0.535)	(0.539)	(0.569)
β_2[d]		0.503	0.006
		(0.530)	(0.029)
Observations	814	814	814
Log Likelihood[e]	-2232.123	-2231.498	-2230.984
Akaike Information Criterion[f]	4474.245	4474.997	4473.968
Bayesian Information Criterion[g]	4497.755	4503.209	4502.180

The dependent variable was 30-MMSE in all models. Time was measured in years. All parameter estimates are presented as mean ± standard error.
[a] Scaling factor for the exponential model.
[b] Scaling factor for time in the exponential model.
[c] Estimated effect of glycan positivity.
[d] Estimated effect of female sex or age (in years).
[e] Higher number indicate better model fit.
[f, g] Lower number indicate better model fit.
* $p < 0.05$, ** $p < 0.01$, *** $p < 0.001$

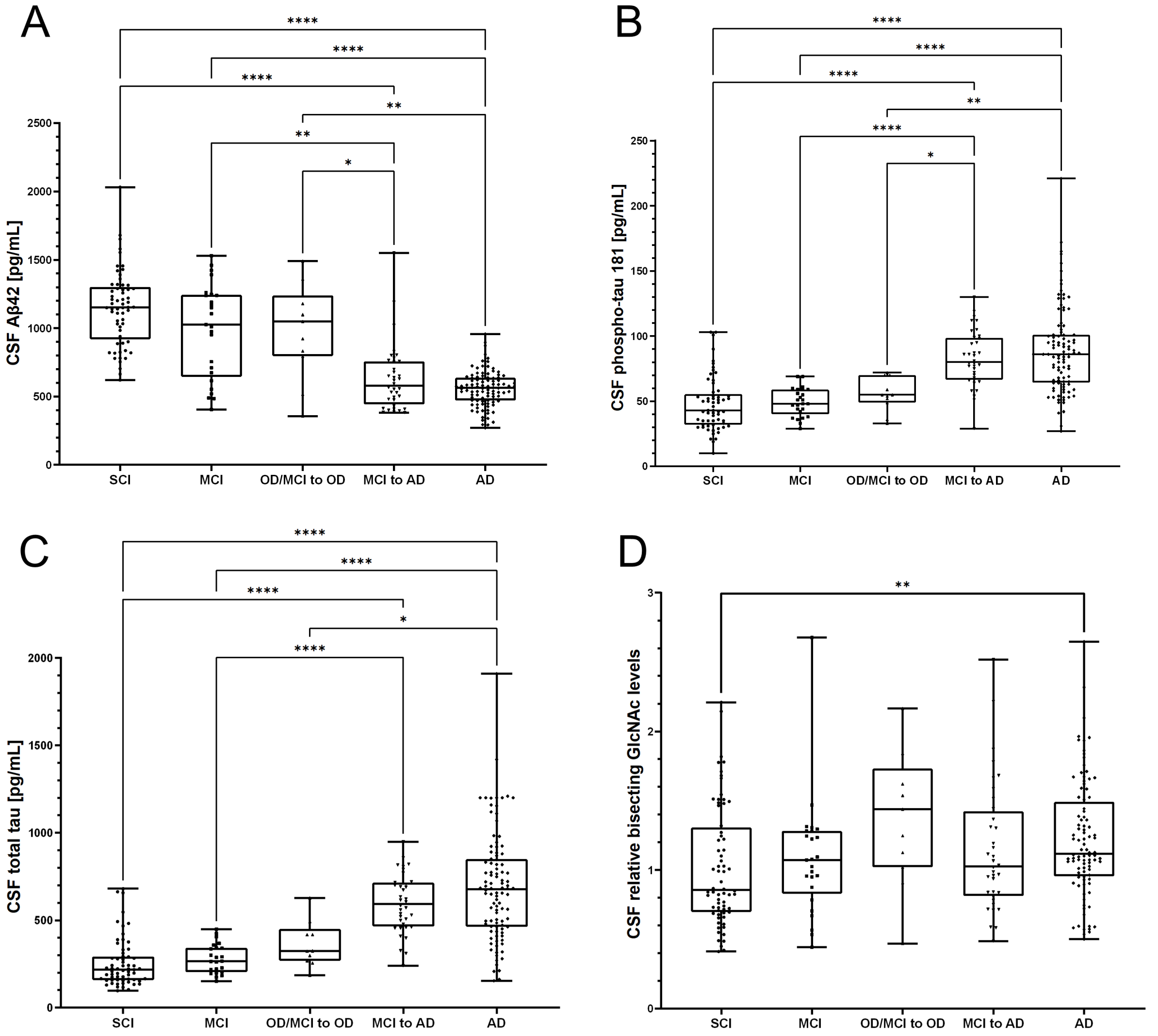

Supplementary Figure 1 Levels of CSF biomarkers at baseline in different disease groups. (A-C) Levels of Aβ42, phosphorylated tau-181, and total tau in CSF. The number of individuals in each group was n = 61 in SCI, n = 25 in MCI, n = 11 in OD/MCI to OD, n = 36 in MCI to AD, and n = 100 in AD. **(D)** Relative levels of bisecting GlcNAc in each disease group. The number of individuals were n = 68 in SCI, n = 25 in MCI, n = 11 in OD/MCI to OD, n = 36 in MCI to AD and n = 96 in AD. In all graphs, whiskers indicate maximum and minimum values, while boxes show median, 25th percentile, and 75th percentile values. Groups were compared using Kruskal Wallis H-test with Dunn's multiple comparisons. Significances shown: *$p < 0.05$, **$p < 0.01$, ***$p < 0.001$, ****$p < 0.0001$. Abbreviations: AD = Alzheimer's disease, MCI= Mild cognitive impairment, MCI to AD = Mild cognitive impairment converting to Alzheimer's disease, MCI to OD = Mild cognitive impairment converting to other dementia, OD = Other dementia, SCI = Subjective cognitive impairment.

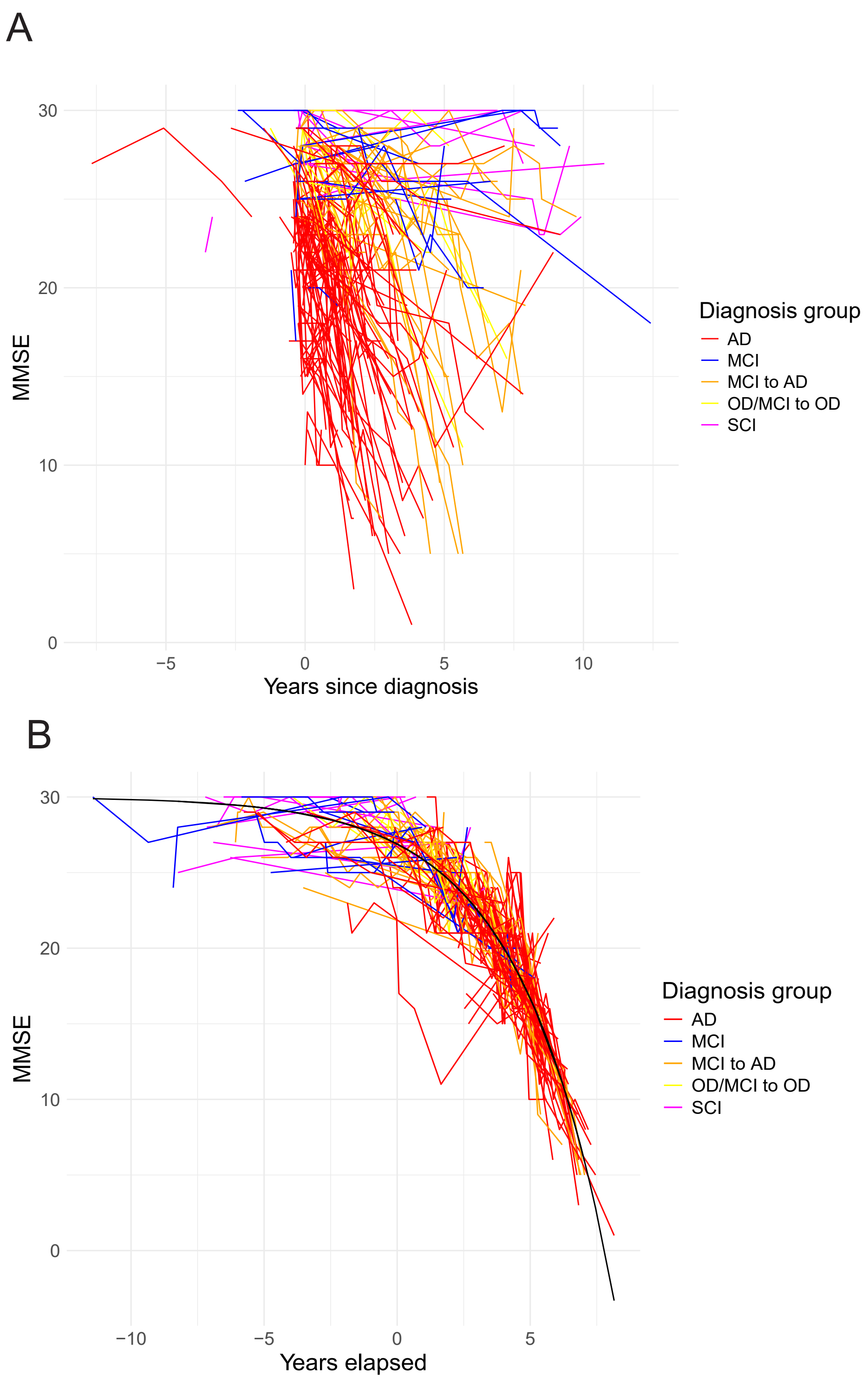

Supplementary Figure 2 Cognitive decline as measured by longitudinal Mini-Mental State Examination (MMSE) scores in the GEDOC cohort. (A) MMSE trajectories of patients in the GEDOC cohort stratified by disease groups. The x-axis refers to time relative to the date of initial diagnosis. **(B)** Mean MMSE trajectory of the GEDOC cohort over time, modelled using a non-linear mixed effects model with individual time shift. Abbreviations: AD = Alzheimer's disease, MCI= Mild cognitive impairment, MCI to AD = Mild cognitive impairment converting to Alzheimer's disease, MCI to OD = Mild cognitive impairment converting to other dementia, MMSE = Mini-Mental State Examination, OD = Other dementia, SCI = Subjective cognitive impairment.

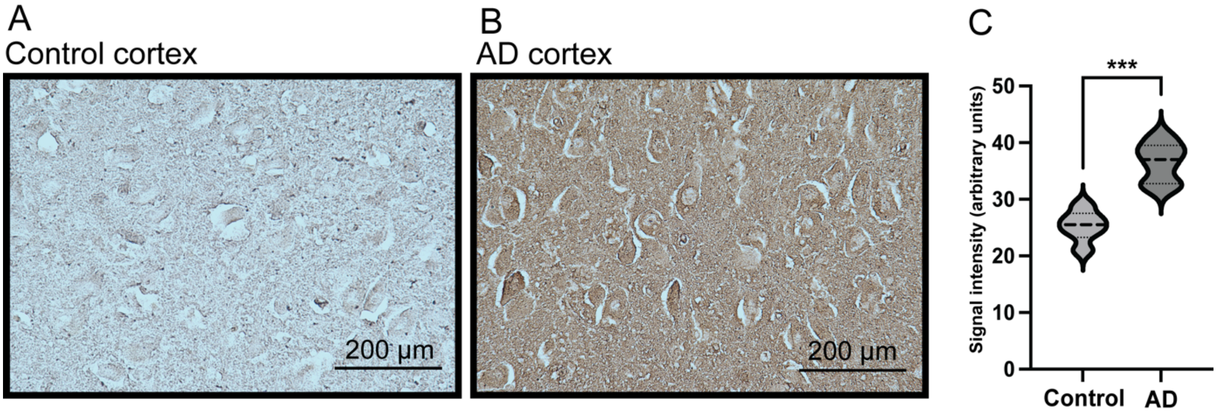

Supplementary Figure 3 Immunohistochemistry of Phaseolus vulgaris erythroagglutinin (PHA-E) in Alzheimer's disease (AD) and control cortex. (A, B) Representative images displaying PHA-E staining in cortex of control and AD brain. **(C)** Quantification of PHA-E signal intensity in cortex of AD compared to control. Graph shows signal intensity with lines indicating median, 25th percentile, and 75th percentile. Groups were compared using unpaired two-tailed Student's t-test. Significance shown: *** $p < 0.001$.

Supplementary Methods: R code used for data analysis and visualization in this study.

```
library(nlme)
library (lme4)
library(readxl)
library(tidyr)
library(ggplot2)
library(dplyr)

#Data
processing--------------------------------------------------------
-------
#Drop NAs
df <- drop_na (data, "mmse")
#Format time as numeric
df <- mutate(df, time=as.numeric(time))
#Change time scale to years
df <- mutate(df, time=time/12)
#Format age as numeric
df <- mutate(df, age=as.numeric(age))
#Transform sex to binary value
df$sex[df$sex == "F"] <- 1
df$sex[df$sex == "M"] <- 0

#Join groups
df$cat[df$cat == "MCI to SCI"] <- "MCI"
df$cat[df$cat == "OD"] <- "OD/MCI to OD"
df$cat[df$cat == "SCI to MCI"] <- "SCI"
df$cat[df$cat == "MCI to OD"] <- "OD/MCI to OD"

#Set colours
custom_colors <- c("red", "blue", "orange", "yellow", "magenta")

#Spaghetti plot
ggplot(aes(time, mmse, group=id, color=factor(cat)), data = df)
+geom_line() +
  scale_color_manual(values = custom_colors) +
  labs(
    title = "",
    x = "Years since diagnosis",
    y = "MMSE",
    color = "Diagnosis group"
  ) +
  theme_minimal() +
  theme(
    text = element_text(size = 18))

#Linear mixed effects model
----------------------------------------------------
lme1 <- lmer(30-mmse ~ time + (1 | id), data = df)
summary (lme1)

#Non-linear mixed model
--------------------------------------------------------

#Fit exponential model
```

```
nlsout <- nls(30-mmse ~ A*exp(alpha*(time)),
start=list(A=2,alpha=0.05),
              data=df,)
nlsout

#Retrieve coefficients to use as starting values for mixed model
start_val<-summary(nlsout)$coefficients[,1]

#Fit non-linear model with individual time shift
nlmeout<-nlme(30-mmse ~ A*exp(alpha*(time+s)),
              fixed=list(A ~ 1,
                         alpha ~ 1),
              random=s ~ 1 | id,
              start=c(start_val),
              data=df)
nlmeout

#Add estimated individual time shifts to original data set

df<-df %>% left_join(

data.frame(id=as.numeric(row.names(nlmeout$coefficients$random$id))
             ,nlmeout$coefficients$random$id), by='id'
) %>%
  #compute shifted time points
  mutate(time_shifted=time+s)

#Add mean predictions
df$pred<-30-predict(nlmeout, newdata=df)

#Spahetti plot with shifted time
ggplot(aes(time_shifted, mmse, group=id, color=factor(cat)),
data=df)+ geom_line() +
  geom_line(aes(x=time_shifted,y=pred, color=NULL, group = cat)) +
  scale_color_manual(values = custom_colors) +
  labs(title = "",
    x = "Years elapsed",
    y = "MMSE",
    color = "Diagnosis group") +
  theme_minimal() +
  theme(text = element_text(size = 18))

#Glycan as fixed effect
analysis---------------------------------------------

#Dichotomize csf bisecting GlcNAc
df <- drop_na (df, "csfpha")
df$csfpha[df$csfpha > 1] <- 1
df$csfpha[df$csfpha < 1] <- 0

#fit simple model
nlsout <- nls(30-mmse ~ A*exp((alpha)*(time+D*csfpha)), data = df,
start=list(A=2, alpha=0.1, D=0))
nlsout
```

```
#Retrieve coefficients to use as starting values for mixed model
start_val<-summary(nlsout)$coefficients[,1]

#Fit non-linear model with individual time shift
nlmeout<-nlme(30-mmse ~ A*exp(((alpha)*(time+s+D*csfpha))),
              fixed=list(A ~ 1,
                         alpha ~ 1,
                         D ~ 1),
              random=s ~ 1 | id,
              start=c(start_val),
              data=df)

summary(nlmeout)

#Add estimated individual time shifts to original data set
df$s<-NULL
df$time_shifted<-NULL
df$pred <- NULL

df<-df %>% left_join(

data.frame(id=as.numeric(row.names(nlmeout$coefficients$random$id))
             ,nlmeout$coefficients$random$id), by='id'
) %>%
  #compute shifted time points
  mutate (time_shifted=time+s)

#Shift order of plotted curves
df$csfpha[df$csfpha == 1] <- 2
df$csfpha[df$csfpha == 0] <- 1
df$csfpha[df$csfpha ==2] <- 0

#add mean predictions
df$pred<-30-predict(nlmeout, newdata=df)

#Spaghetti plot with shifted time
ggplot(aes(time_shifted, mmse, group=id,
color=factor(csfpha)),data=df)+
  geom_line()+
  #add mean prediction by glycan status
  geom_line(aes(x=time_shifted,y=pred, color=NULL,group=csfpha)) +
  #Add labels
  labs (x="Years elapsed",
        y = "MMSE", color = "")+
  theme_gray(base_size = 30)+
  scale_color_discrete(labels=c('Bisecting GlcNAc positive',
'Bisecting GlcNAc negative'))+
  xlim(-10, 10)+
  ylim(0, 30) +
  theme(legend.position = "top")

#Tau negative or positive
subgroups-------------------------------------------------
```

```
taupos <- subset (df, ptau > 56.5)
tauneg <- subset (df, ptau < 56.5)
#Drop NAs
taupos <- drop_na (taupos, "ptau")
tauneg <- drop_na (tauneg, "ptau")

#Fit simple non-linear model (Tau positive)
nlsout <- nls(30-mmse ~ A*exp((alpha)*(time+D*csfpha)), data =
taupos, start=list(A=2, alpha=0.1, D=0))

#Retrieve coefficients to use as starting values for mixed model
start_val<-summary(nlsout)$coefficients[,1]

nlmeout<-nlme(30-mmse ~ A*exp(((alpha)*(time+s+D*csfpha))),
              fixed=list(A ~ 1,
                         alpha ~ 1,
                         D ~ 1),
              random=s ~ 1 | id,
              start=c(start_val),
              data=taupos)

summary(nlmeout)

#Add estimated individual time shifts to original data set
taupos$s<-NULL
taupos$time_shifted<-NULL
taupos$pred<-NULL

taupos<-taupos %>% left_join(

data.frame(id=as.numeric(row.names(nlmeout$coefficients$random$id))
             ,nlmeout$coefficients$random$id), by='id'
) %>%
  #compute shifted time points
  mutate (time_shifted=time+s)

#Add mean predictions
taupos$pred<-30-predict(nlmeout, newdata=taupos)

#Plot graph
ggplot(aes(time_shifted, mmse, group=id,
color=factor(csfpha)),data=taupos)+
  geom_line()+
  #add mean prediction by dementia status
  geom_line(aes(x=time_shifted,y=pred, color=NULL,group=csfpha)) +

  #Add labels
  labs (x="Years elapsed",
        y = "MMSE", color = "",
        title = "Tau positive")+
  xlim(-10, 10)+
  ylim(0, 30)+
  theme_gray(base_size = 35)+
  scale_color_discrete(labels=c('Bisecting GlcNAc positive',
```

```
'Bisecting GlcNAc negative'))+
  theme(legend.position = "top")

#Fit non-linear model (Tau negative)
nlsout <- nls(30-mmse ~ A*exp((alpha)*(time+D*csfpha)), data =
tauneg, start=list(A=2, alpha=0.1, D=0))

#Retrieve coefficients to use as starting values for mixed model
start_val<-summary(nlsout)$coefficients[,1]

nlmeout<-nlme(30-mmse ~ A*exp(((alpha)*(time+s+D*csfpha))),
              fixed=list(A ~ 1,
                         alpha ~ 1,
                         D ~ 1),
              random=s ~ 1 | id,
              start=c(start_val),
              data=tauneg)

summary(nlmeout)

#Add estimated individual time shifts to original data set
tauneg$s<-NULL
tauneg$time_shifted<-NULL
tauneg$pred<-NULL

tauneg<-tauneg %>% left_join(

data.frame(id=as.numeric(row.names(nlmeout$coefficients$random$id))
             ,nlmeout$coefficients$random$id), by='id'
) %>%
  #compute shifted time points
  mutate (time_shifted=time+s)

#add mean predictions
tauneg$pred<-30-predict(nlmeout, newdata=tauneg)

#Plot graph
ggplot(aes(time_shifted, mmse, group=id,
color=factor(csfpha)),data=tauneg)+
  geom_line()+
  #add mean prediction by dementia status
  geom_line(aes(x=time_shifted,y=pred, color=NULL,group=csfpha)) +

  #Add labels
  labs (x="Years elapsed",
        y = "MMSE", color = "",
        title = "Tau negative")+
  xlim(-10, 10)+
  ylim(0, 30)+
  theme_gray(base_size = 35)+
  scale_color_discrete(labels=c('Bisecting GlcNAc positive',
'Bisecting GlcNAc negative'))+
  theme(legend.position = "top")
```

```
#Amyloid negative or positive
subgroups---------------------------------------------------
amypos <- subset (df, baml42 < 599)
amyneg <- subset (df, baml42 > 599)
#Drop NAs
amypos <- drop_na (amypos, "baml42")
amyneg <- drop_na (amyneg, "baml42")

#Fit non-linear model (Amyloid positive)
nlsout <- nls(30-mmse ~ A*exp((alpha)*(time+D*csfpha)), data =
amypos, start=list(A=2, alpha=0.1, D=0))

#Retrieve coefficients to use as starting values for mixed model
start_val<-summary(nlsout)$coefficients[,1]

nlmeout<-nlme(30-mmse ~ A*exp(((alpha)*(time+s+D*csfpha))),
              fixed=list(A ~ 1,
                         alpha ~ 1,
                         D ~ 1),
              random=s ~ 1 | id,
              start=c(start_val),
              data=amypos)

summary(nlmeout)

#Add estimated individual time shifts to original data set
amypos$s<-NULL
amypos$time_shifted<-NULL
amypos$pred<-NULL

amypos<-amypos %>% left_join(

data.frame(id=as.numeric(row.names(nlmeout$coefficients$random$id))
             ,nlmeout$coefficients$random$id), by='id'
) %>%
  #compute shifted time points
  mutate (time_shifted=time+s)

#add mean predictions
amypos$pred<-30-predict(nlmeout, newdata=amypos)

#Plot graph
ggplot(aes(time_shifted, mmse, group=id,
color=factor(csfpha)),data=amypos)+
  geom_line()+
  #add mean prediction by dementia status
  geom_line(aes(x=time_shifted,y=pred, color=NULL,group=csfpha)) +

  #Add labels
  labs (x="Years elapsed",
        y = "MMSE", color = "",
        title = "Amyloid positive")+
  xlim(-10, 10)+
  ylim(0, 30)+
```

```
  theme_gray(base_size = 35)+
  scale_color_discrete(labels=c('Bisecting GlcNAc positive',
'Bisecting GlcNAc negative'))+
  theme(legend.position = "top")

#Fit non-linear model (Amyloid negative)
nlsout <- nls(30-mmse ~ A*exp((alpha)*(time+D*csfpha)), data =
amyneg, start=list(A=2, alpha=0.1, D=0))

#Retrieve coefficients to use as starting values for mixed model
start_val<-summary(nlsout)$coefficients[,1]

nlmeout<-nlme(30-mmse ~ A*exp(((alpha)*(time+s+D*csfpha))),
              fixed=list(A ~ 1,
                         alpha ~ 1,
                         D ~ 1),
              random=s ~ 1 | id,
              start=c(start_val),
              data=amyneg)

summary(nlmeout)

#Add estimated individual time shifts to original data set
amyneg$s<-NULL
amyneg$time_shifted<-NULL
amyneg$pred<-NULL

amyneg<-amyneg %>% left_join(

data.frame(id=as.numeric(row.names(nlmeout$coefficients$random$id))
             ,nlmeout$coefficients$random$id), by='id'
) %>%
  #compute shifted time points
  mutate (time_shifted=time+s)

#add mean predictions
amyneg$pred<-30-predict(nlmeout, newdata=amyneg)

#Plot graph
ggplot(aes(time_shifted, mmse, group=id,
color=factor(csfpha)),data=amyneg)+
  geom_line()+
  #add mean prediction by dementia status
  geom_line(aes(x=time_shifted,y=pred, color=NULL,group=csfpha)) +

  #Add labels
  labs (x="Years elapsed",
        y = "MMSE", color = "",
        title = "Amyloid negative")+
  xlim(-10, 10)+
  ylim(0, 30)+
  theme_gray(base_size = 35)+
  scale_color_discrete(labels=c('Bisecting GlcNAc positive',
'Bisecting GlcNAc negative'))+
```

```
theme(legend.position = "top")
```
